# Compound Fracture of the Lower Extremity

**Published:** 1893-11

**Authors:** R. Menger

**Affiliations:** San Antonio, Texas


For Texas Medical Journal.

### COMPOUND FRACTURES OF LOWER EXTREMITY.

R. MENGER, M. D., SAN ANTONIO, TEXAS.

Read before the Annual Meeting of the West Texas Medical Association.

WE ARE all aware of the importance and difficulty of treating fractures of the lower extremities, especially compound fractures, and more so of fractures involving the femoral bone. More or less difficulty is experienced in the immobilization of the extremity, be it by means of splints, extension, and counter-extension, plaster-bandages, fracture-boxes, etc., the main trouble encountered in such cases being the inconvenience to the patient during stool. If I am not mistaken, some German surgeon is the inventor of using a long splint, or rather a broad board, in cases of femoral and other fractures of the lower extremities. This board reaches from below the shoulders down to and above the heel of the foot, and should be so arranged that the patient lays at the gluteal region, on a hollow, made by cutting a circular piece out of the board. At the site of the fracture the board can be converted into a fracture-box, with or without a door, adjusted by hinges, in order to investigate the condition of the fractured part at any time. When the patient intends to move his bowels, he is lifted on this fracture-board by lifting the board near his shoulders, and a bed-pan can then be adjusted under him without much or no disturbance to the patient and his broken limb. Of course, the board should be well padded, and an oilcloth spread on its entire length, to avoid, if possible, pressure sores, and to keep the bedding clean, in case drainage, etc., is used. Still, even with this appliance, some difficulty and inconvenience may be encountered, especially in compound fractures of the femur, complicated with lacerations of the cuticle and deeper tissues. Compound fractures of the tibia and fibula, even when complicated with extensive lacerations of the soft parts, can be handled by such a board somewhat easier, after the limb is properly encased in plaster of Paris, etc. According to the nature of the case, especially in compound fractures, the precaution should always be kept in view to resect the fracture-ends, or smooth them with the bone-forceps, in case the edges are splintered or rough; and it is interesting to note to how much extent the loss of bone-substance is tolerated and regenerated, even in spite of much bone-missing or severe lacerations of the periosteum and soft parts. A case of compound fracture of the lower extremity, in a very heavy man, will be recollected by Dr. Berry, in which we were induced to resect fully 2½ inches of the entire bone-substance of the tibia on account of severe splintering of the fracture-ends, and, after encasing the lacerated leg iu a well-padded plaster of Paris bandage, with an opening at the site of the fracture, the space of missing bone was restored by osseous tissue in a comparative short time, so that, after the second month, the man was able to stand and walk without a crutch, and he never experienced any trouble afterward, and there was but very little shortening of his leg.

A similar and even more serious case occurred in my practice last year, when a German boy, aged 9 years, was brought here, a distance of about twenty-two miles, with a compound fracture of the left femoral bone, with extensive lacerations of the skin and deeper soft parts. The accident occurred in the following manner: His parents were going to a pic-nic, and whilst the ambulance started the boy was behind and on the vehicle, and by some means he slipped and his left leg got caught between the spokes of the wheel which, during a turn, broke the femoral bone in the lower third of the thigh, horribly lacerating the soft parts. When sent here his clothing was saturated with blood, and the broken bone had penetrated his pants. After removing his clothing the upper fracture end was protruding fully three inches through the lacerated muscles, and the wide open lacerated places reached from the summit to the base of the thigh. It was deemed necessary to resect the splintered fracture-edges of both ends, and Drs. Berry and A. Graves, jr., assisted during the operation. Ether and whiskey injections had to be made repeatedly, on account of the very prostrated condition of the boy. He was then put comfortably in bed with hot bottles to his side and two large sand-bags along both sides of his leg, the outer one reaching upward near the shoulder. He was chloroformed again after the second day, and the leg encased in a plaster of paris bandage, which although, after the end of the first week had to be removed on account of severe complaint of the boy, over the suppurating places around and below the knee joint where the skin had been torn off by the injury. He was now, after chloroforming again, put on a fracture board, similar to the one described in the beginning of this paper. A large opening or door with hinges to the board had been provided on the fracture-box at the site of the injury, and the wound thus could easily be washed and dressed with aristol ointment. At the end of the fifth week this fracture box had also to be dispensed with on account of the bran becoming offensive and impregnated with the wound secreta, and as ossification by this time was well established, the leg was only kept quiet by sandbags. After about seven weeks this case was discharged, as the boy could safely be transported to his home on a ranch,—luckily for him, as the same night after he was removed, the room of the upper story in which he was lying, caught fire and burned up. I will now, gentlemen, take pleasure in showing you the boy's injuries, and you will notice how little the leg has shortened, considering the extensive lacerations and the amount of bone lost during the injury and resection. He limps but little, and has not worn a crutch for the last seven months. But there still is a small fistulous opening at the site of fracture discharging pus, indicating slight superficial osteo-necrosis—which I intend to correct to-morrow. I will also show you some illustrations of this case, showing the extent of the protruding fractured femoral bone at the time the boy was brought here, also the resected bone ends, the granulating surface and the injured leg when nearly healed.

